
JOURNAL INFORMATION
==============================
NLM Title Abbreviation: ScientificWorldJournal
Journal ID: ScientificWorldJournal
NLM Journal ID: 101131163
Journal ID: TSWJ

The Scientific World Journal

ISSN: 2356-6140
EISSN: 1537-744X
Publisher: Wiley

ARTICLE INFORMATION
==============================
PMCID: PMC6084706
PMID: 30147501
DOI: 10.1100/tsw.2001.189
Article ID: 826549
Article version: 1
Subjects: Short Report

Stress and the Control of Apoptosis

Green Douglas R.
La Jolla Institute for Allergy and Immunology, 10355 Science Center Dr., San Diego, CA 92121, USA
Publication date: 2001
Electronic publication date: 2001 Jun 8
Volume: 1
First page: 47
Last page: 47
Copyright: Copyright © 2001 Douglas R. Green.
Copyright year: 2001
License: This is an open access article distributed under the Creative Commons Attribution License, which permits unrestricted use, distribution, and reproduction in any medium, provided the original work is properly cited.
License URL: https://creativecommons.org/licenses/by/3.0/

==============================
Miami *Nature Biotechnology* Short Reports
*TheScientificWorld* (2001) 1 (S3), 47SR
ISSN 1532-2246; DOI 10.1100/tsw.2001.189

### STRESS AND THE CONTROL OF APOPTOSIS

Douglas R. Green

La Jolla Institute for Allergy and Immunology, 10355 Science Center Dr., San Diego, CA 92121
dgreen5240@aol.com

Apoptotic cell death is coordinated by the caspase proteases, and these are activated via two major pathways. One of these depends upon the binding of death receptors, such as Fas (CD95) by their ligands. Alternatively, apoptosis can proceed by the activation of pro-apoptotic Bcl-2 family proteins that induce a permeabilization of the mitochondrial outer membrane, releasing proteins from the intermembrane space. Cytochrome c then triggers caspase activation. In either case, activated "executioner" caspases cleave key substrates within the cell that then cause the apoptotic death. Not all of these key substrates are known.

Cellular stress can engage either or both of these pathways to cause apoptosis. DNA damage, heat shock, and other stressors induce expression of Fas-ligand (FasL) on several different cell types, and can sensitize cells to death by ligation of Fas. Dissection of the FasL promoter reveals a number of novel regulatory features consistent with such stress-induced expression. For example, both AP-1 and NF-κB are activated following DNA damage, and this activation is required for induction of FasL expression via binding of these transcription factors to their sites in the FasL promoter. In addition, the promoter appears to be regulated by c-Myc/Max heterodimers, suggesting a link to activation of this important proto-oncogene. We have identified a noncanonical Myc/Max binding site within the FasL promoter that appears to be responsible for this effect. We confirmed that this is indeed a Myc-responsive element by transplanting this site to the promoter for ornithine decarboxylase, replacing its canonical c-Myc-binding sites. The chimeric promoter became much more responsive to c-Myc than even the original ODC promoter. Conversely, mutation of the c-Myc site in the FasL promoter destroys its responsiveness to c-Myc or to other activating signals. Therefore, c-Myc is likely to be an important component of FasL expression. When FasL is expressed, it engages Fas on either the same or a neighboring cell. This can include infiltrating inflammatory cells, and the FasL may serve to protect the tissue from inflammatory damage. Upon ligation of Fas, caspases are activated and the cell dies.

Cellular stress also induces mitochondrial outer membrane permeabilization. For example, activation of p53 induces the translocation of the pro-apoptotic Bcl-2 family protein Bax from the cytosol to the mitochondria, resulting in cytochrome c release, caspase activation, and death. A region of p53 required for this effect is distinct from known transactivation, DNA binding, or tetramerization domains. Intriguingly, it is possible that not all of the effects of p53 in inducing apoptosis require transcription.

In cells subjected to stress, cytochrome c is released suddenly following a variable time lag, and this release is complete within 5 minutes. Apoptotic events dependent upon caspase activation then occur on schedule. Although the release of cytochrome c appears to be coordinated throughout the cell, this does not appear to involve intermitochondrial communication, since decreasing the temperature (which greatly slows down phenomena such as the mitochondrial permeability transition) does not affect the 5- minute interval from first to last mitochondrion to release in a cell.

Although the release of cytochrome c should result in a disruption of the mitochondrial electron transport chain, this does not occur. Single cell analysis of mitochondrial change during cytochrome c release show only a transient loss of inner membrane potential, after which there is a compensation such that the reduced levels of cytochrome c are at least temporarily sufficient for maintenance of electron transport and ATP generation. Upon caspase activation, mitochondrial function is lost.

Stress can also prevent cell death. Cells under stress produce high levels of Hsp70, which protects cells and leaves them resistant to subsequent stress. One effect of Hsp70 is to make cells resistant to apoptosis, and one way that this occurs is downstream of the mitochondria. Cytochrome c acts to trigger the oligomerization of Apaf-1, a molecule that then binds procaspase-9 and activates it. This in turn activates other caspases and kills the cells. Hsp70 prevents the recruitment of procaspase-9 to the “apoptosome” thus preventing caspase activation.

**SELECTED FURTHER READING.**

Green, D.R. (1998) Cell 94, 695-698

Green, D.R. (2000) Cell 102, 1-4

Kasibhatla, S., Brunner, T., Genestier, L., Echeverri, F., Mahboubi, A., and Green, D.R. (1998) Mol. Cell 1, 543-551

Kasibhatla, S., Beere, H.M., Brunner, T., Echeverri, F., and Green, D.R. (2000) Curr. Biol. 10, 1205-1208

Schuler, M., Bossy-Wetzel, E., Goldstein, J.C., Fitzgerald, P., and Green, D.R. (2000) J. Biol. Chem. 275, 7337-7342

Goldstein, J.C., Waterhouse, N.J., Juin, P., Evan, G.I., and Green, D.R. (2000) Nat. Cell Biol. 2, 156-162

Beere, H.M., Wolf, B.B., Mosser, D.D., Cain, K., Kuwana, T., Morimoto, R.I., Cohen, G.M., and Green, D.R. (2000) Nat. Cell Biol. 2, 469-475
